# Early Response and Clinical Efficacy of a Mouthwash Containing Chlorhexidine, Anti Discoloration System, Polyvinylpyrrolidone/Vinyl Acetate and Sodium DNA in Periodontitis Model: A Triple-Blind Randomized Controlled Clinical Trial

**DOI:** 10.3390/dj10060101

**Published:** 2022-06-07

**Authors:** Felice Lorusso, Gianluca Tartaglia, Francesco Inchingolo, Antonio Scarano

**Affiliations:** 1 Department of Innovative Technologies in Medicine and Dentistry, University of Chieti-Pescara, 66100 Chieti, Italy; felice.lorusso@unich.it; 2 Department of Biomedical, Surgical and Dental Sciences, School of Dentistry, University of Milan, 20122 Milan, Italy; gianluca.tartaglia@unimi.it; 3 Department of Interdisciplinary Medicine, University of Medicine Aldo Moro, 70124 Bari, Italy; francesco.inchingolo@uniba.it; 4 Department of Oral Implantology, Dental Research Division, College Ingà, UNINGÁ, Cachoeiro de Itapemirim 29312, Brazil

**Keywords:** periodontitis, plaque, oral hygiene, inflammation, bacteria, biofilms

## Abstract

Polyvinylpyrrolidone/vinyl acetate (PVP/VA) is a molecule with increased adhesion capacity, and can be associated in the bacterial plaque control with sodium DNA, a natural anti-aging molecule able to improve gingival trophism. The aim of the study is to test at two weeks the antimicrobial and antiplaque properties, subjects affected by chronic periodontitis, showed by a mouthwash containing Chlorhexidine (CHX) 0.2% with Anti Discoloration System (ADS), PVP-VA, and Sodium DNA in comparison with a placebo mouthwash. A single center randomized controlled trial was conducted on a total of fifty-four (54) subjects. In the test Group (*n* = 27) patients were treated by a 0.2% Chlorhexidine (CHX) mouthwash with ADS, PVP-VA, and Sodium DNA, while a placebo mouthwash was used in the control Group (B). The full mouth plaque score (FMPS), full mouth bleeding score (FMBS), and gingival index (GI) were assessed at baseline, and at 1 and 2 weeks after treatment. FMPS score recorded at baseline (V2) was 52.7 ± 9.2 in the Group Test and 58.2 ± 6.1 in the Group Control (*p* > 0.05). After 1 week (V3), FMPS was 13.3 ± 5.6 in the Group Test and 18.7 ± 4.3 in the Group Control (*p* < 0.05), while at V4 (2 weeks), FMPS was 14.2 ± 4.1 in the Group Test and 20.3 ± 5.2 in the Group Control (*p* < 0.05). FMBS score recorded at baseline (V2) was 46.7 ± 8.7 in the Group Test and 49.2 ± 6.2 in the Group Control (*p* > 0.05). After 1 week (V3), FMBS was 12.7 ± 4.2 in the test Group Test and 18.5 ± 5.9 in the control Group Control (*p* < 0.05), while after 2 weeks (V4), it was 13.1 ± 3.2 in the Group Test and 19.8 ± 4.9 Group Control (*p* < 0.05). This trial has clinically showed the efficacy of a new formulation of chlorhexidine mouthwash in reducing bacterial plaque and gingival inflammation. A significant reduction of inflammation and bleeding scores was found in periodontal patients treated by a mouthwash containing CHX 0.2% with ADS, PVP-VA, and Sodium DNA compared to those treated with a placebo mouthwash.

## 1. Introduction

Proper plaque control is essential for oral health maintenance and to reduce the risk of periodontal diseases development [1]. Adequate antibacterial control is also essential during peri- and post-operative management in patients subjected to oral dental procedures to obtain optimal healing responses without infectious processes [2,3,4,5]. The aggregation of bacterial biofilms is a physiological process that takes place in the very early stages after the usual oral hygiene maneuvers, through the primary colonization of Gram-positive bacteria, such as streptococci, and fungi could produce a progressive maturation that involves an increase in the population of Gram-negative and potentially pathogenic microorganisms such as fusobacteria and spirochete [6,7,8]. Torkzaban et al., reported that the flossing followed by the toothbrushing is able to produce the optimal plaque control with a reduction of the clinical bleeding point index (BPI) [9]. Moreover, others interdental cleaning aids such as rubberpicks/wooden do not demonstrate inferiority to conventional devices, while irrigators are able to reduce gingival inflammation for natural teeth and dental implants maintenance [10].

Such mechanical hygienic maneuvers are, however, often affected in patients undergoing surgical treatment or in subjects with poor compliance and manual ability; in these cases the associated use of chemical agents, such as antibacterial mouthwashes, in conjunction with mechanical brushing is necessary [11]. To date, 0.2% CHX has revealed the greatest effectiveness of plaque control, due to the prolonged duration of action within the oral cavity as well as the adsorption capacity on hard surfaces and prolonged release [12]. The most common side effect of the 0.2% CHX concentration is represented by brownish pigmentations that occur on dental surfaces, restorations, dental prostheses, and the tongue [13]. In the literature, others main side effects correlated to CHX-based mouthwash formulation were taste alteration during treatment and higher tartar formation, especially in prolonged administration over 14 days [14,15,16,17]. This effect, undermining the compliance of the patient, often limits its use [2]. The Anti Discoloration System (ADS) allows to combine antiplaque activity with aesthetic function, reducing the tooth enamel pigmentation without reducing the CHX effectiveness [18]. The film-forming property of Polyvinylpyrrolidone/vinyl acetate (PVP-VA) allows the formation of a protective interface that protect the underlying tissues from bacterial or chemical attacks. The film-forming properties of these polymers are able to protect the dental surfaces or oral mucosal membranes even if not intact are known [7].

Claydon et al., investigated the role of chlorhexidine in a PVP-VA/CHX association produce an adjuvant function; in fact, it protects the film-forming layer from contamination or bacterial over-infection, prolongs its permanence with no direct effect on tissues and wounds, as limited by the mechanical film layer barrier [19]. Therefore, it is demonstrated that the presence of PVP-VA in mouthwash solution or in a periodontal gel is able, alone, to ensure antibacterial protection [19]. The sodium DNA is a natural molecule widely used in cosmetics as an anti-aging product [20]. The sodium DNA is a xenogenic component characterized by a biological and functional activity; in vitro findings showed that this compound is able to stimulate the cell activity though a growth factors and cytokines modulations [21].

The aim of the study was to test, at two weeks, the antimicrobial and antiplaque control, of a mouthwash containing 0.2% CHX/ADS, PVP-VA and Sodium DNA compared to a placebo mouthwash on subjects affected by periodontitis.

## 2. Materials and Methods

### 2.1. Trial Study Design

The study was conducted in accordance with the Helsinki Declaration, the Good Clinical Practice Guidelines for clinical trials on medicinal products (GCP E6 R2:2017) and UNI EN ISO 14155:2017 and approved by the Ethical Committee for Biomedical Research of Chieti and Pescara (N°1853, 5 December 2019). The study was designed as a randomized single-center controlled, triple-blind trial with two parallel groups. There were no changes after trial commencement. The trial, lasting 2 weeks, was conducted on a total of fifty-four (54) subjects. In the Test Group (*n* = 27), patients were treated by a 0.2% CHX with ADS, PVP-VA, and Sodium DNA mouthwash (Curasept S.p.A., Saronno, Italy), while 27 patients were submitted for a placebo mouthwash treatment as control Group (B). All subjects were instructed to the correct oral hygiene procedures using a chlorhexidine-free toothpaste deferred from the administration of the mouthwash solution.

### 2.2. Mouthwash Solution Composition

The tested mouthwash formulation (Curasept Spa, Saronno, Italy) was composed of a solution of 0.2% Chlorexidine Anti Discoloration System (ADS), PVP-VA, and Sodium DNA. The ADS system is composed of ascorbic acid and sodium metabilsulfate, while the adding of 0.12% polyvinylpyrrolidone/vinyl acetate has proven their efficacy in avoiding the chlorexidine capability to bind the surface of the tooth and the soft tissue membranes, and their pigmentation maintaining the anti-plaque efficacy. The 0.2% of Sodium DNA Sodium DNA (Na-DNA) is a biologically active, functional compound obtained by deoxyribonucleic acid of the gonadic tissue of male sturgeons. These molecules submitted a purification, de-polymerization process and finally neutralization sodium (Na^+^) ions. The PVP-VA polymer associated with with sodium DNA improve the gingival tissues trophism and improve their effect on bacterial plaque control. The placebo mouthwash was composed by an aqueous alcohol solution (Betafarma, Cesano Boscone, Italy).

### 2.3. Participants and Eligibility Criteria

Patients were recruited according to the following inclusion criteria:

- Patients with periodontitis with PD > 3 mm in more than or equal to 20 elements (Stage III) [22];
- Non-smokers or moderately smokers (<10 cigarettes/day);

The exclusion criteria were as follows:

- Patients with orthodontic devices;
- Intolerances or allergies to mouthwash;
- Smoking (>10 cigarettes/day) and consumption of alcohol;
- Patients undergoing radiant therapy/chemotherapy for less than 5 years;
- Immunosuppressed patients;
- Systemic, renal or cardiovascular diseases;
- Pregnant, breastfeeding women or patients undergoing antibiotic and anti-inflammatory therapy.

### 2.4. Allocation Procedure

A simple randomization was performed using a dedicated Randomizer application (Urbaniak, G. C., & Plous, S. (2013). Research Randomizer (Version 4.0) [Computer software]. http://www.randomizer.org/, accessed on 22 June 2013) capable of generating random numerical alpha sequences for the patient’s allocation in the respective treatment groups Test and Control. Blinding of participants, data collectors, and data analysts was applied. Operators and patients were not informed about the mouthwash content: the blinded mouthwashes had a similar packaging, with no signs or references that could indicate their formulation. Mouthwashes were provided to patients after instruction on how to use the home, rinse for one minute, away from brushing performed twice a day, every 8 h (20 mL for each administration).

### 2.5. Clinical Evaluation and Data Recording

**Figure 1:**
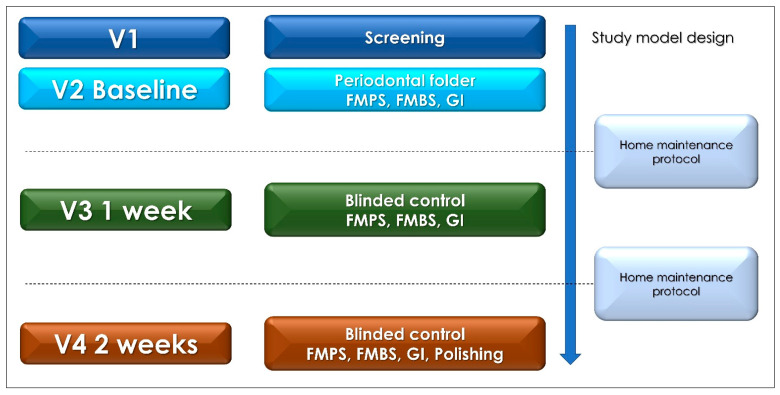
Clinical Trial and study design flowchart (V1, First visit; V2, baseline visit, V3, 1 week visit; and V4, 2 weeks visit).

The clinical evaluation by recording the levels of plaque, bleeding on probing, and depth of pocket were carried out by a single operator and recorded according to the assigned codes (A, B) (Figure 1). The data assessment was performed by two blinded standard examiners, that is, well-trained, experienced, specialist clinicians, in order to reduce the measurement bias. A screening visit to assess the eligibility of the patient and inclusion in the study was performed (**V1**) (Figure 1). Phase V1 provided for the recruitment of subjects and an informed consent was also approved at this stage and instructions for oral hygiene were provided. At the second clinical visit (**V2**), a periodontal folder with Full Mouth Plaque Score (FMPS) and Full Mouth Bleeding Score (FMBS), Gingival Index (GI), and pocket depth registration was performed. Supragingival calculus removal employing ultrasonic scaler tips and accurate polishing with prophylaxis pastes were also performed in this session after the recordings. Patients received the mouthwash package in relation to the assigned study group (A or B) and were invited to perform a first rinse with 10 mL of mouthwash. The protocol provided for a total of three home rinses per day for 2 weeks of treatment. After 1 (**V3**) and 2 weeks (**V4**), a control visit and a new recording of the, FMPS, FMBS, GI, and complications was performed (Figure 1). At **V4** timepoint, a novel clinical evaluation with a final polishing refinement was performed (Figure 1). In phase V2, the periodontal folder with collection of FMPS, FMBS, and GI and radiographic collection was realized, as per clinical protocol. The first 1-min administration of 10 mL of mouthwash was carried out during session V2 in the presence of the operator. Patients were asked to rinse with the assigned mouthwash of one minute, to be performed twice a day for the next two weeks. In phase V3 and V4, the recording study indices such as FMPS, FMBS and GI was carried out again.

### 2.6. Study Outcomes

The following clinical parameters has been evaluated in the present investigation:

− FMPS: the presence/absence of plaque has been recorded in four sites per tooth and the percentage is calculated in relation to the surfaces. The results have been reported as percentage of the positive sites [23];
− FMBS: the presence/absence of bleeding has been recorded at four sites per tooth and the percentage is calculated in relation to the surfaces. The results have been reported as percentage of the positive sites [23];
− Gingival Index (Loe &Silness 1963): the health of periodontal tissues have been recorded according to the following criteria [24]:

0 = Normal gingiva;
1 = Mild inflammation and slight edema and color change in the absence of bleeding on probing;
2 = Moderate inflammation- reddened tissues, edematosis, and bleeding at the test;
3 = Severe inflammation marked redness, edema, ulceration, and tendency to bleeding.

### 2.7. Sample Size Calculation

The sample size calculation was performed by a dedicated software package GPOWER (http://www.gpower.hhu.de/, accessed on 1 January 2019). In the present study, a total of 2 study groups (Test and Control) were considered (α error: 0.05; 80% power). Therefore, for the purposes of statistical significance of the study for *p* < 0.05, a sample size of 54 patients is estimated with an enrollment ratio between the two groups of 1:1 (27 patients per experimental group) [19,20].

### 2.8. Statistical Analysis

The study data were statistically evaluated with the software package Graphpad 6 (Prism, San Diego, CA, USA). The descriptive statistics was performed considering the means, standard deviations and the 95% confidence intervals of the study groups at the different study timepoints. The Bland–Altman plot was applied to evaluate the inter-class agreement and reliability of the study data. The parametric Pearson and non-parametric Spearman correlation tests were applied respectively for FMPS, FMBS, and GI for this purpose.

The normality distribution of FMPS, FMBS, and GI data for experimental groups at different experimental times, such as baselines, 1 week, and 2 weeks, were evaluated by Kolmogorov–Smirnov Test. The significance of FMPS and FMBS data was evaluated by One-way ANOVA followed by Tukey’s post-hoc test. The GI data were assessed by the Kruskal–Wallis test followed by Dunn’s multiple comparison test. The level of significance was considered for *p <* 0.05.

## 3. Results

### 3.1. Main Characteristics of the Study Population

A total of 54 subjects were included in this trial and treated, 27 in the Test group and 27 in the Placebo group. No drop out cases were reported during the investigation and at the end of the trial. The distribution of the pocket probing depth of the Test and Control Group at the baseline and after 2 weeks of treatment has been presented in Table 1.

The average age of enrolled patients was 42.5 ± 7.2 years, consisting of (53.7%) women (29) and 46.3% men (25) (Table 2).

**Figure 2:**
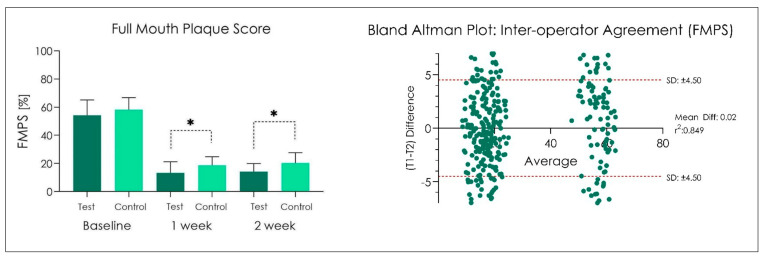
FMPS measurements at baseline, after 1 and 2 weeks. * *p* < 0.05. One-way ANOVA followed by Tukey’s post-hoc test.

**Figure 3:**
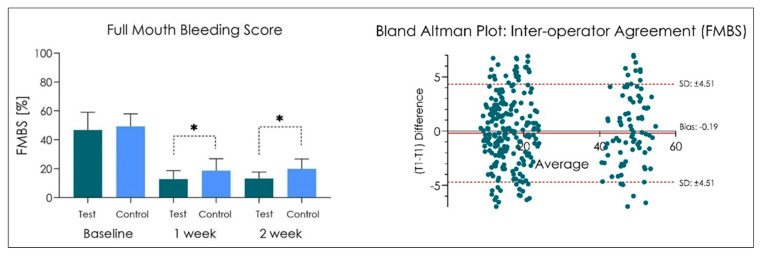
FMBS measurements at baseline, after 1 and 2 weeks. * *p* < 0.05. One-way ANOVA followed by Tukey’s post-hoc test.

**Figure 4:**
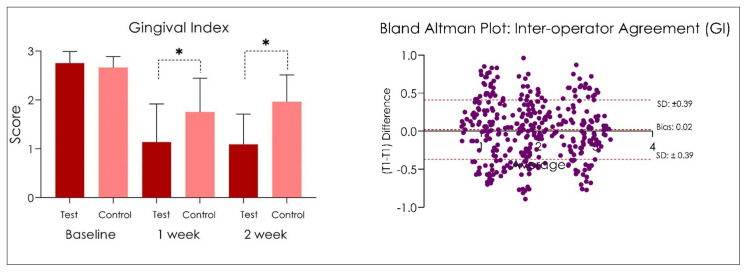
Gingival index score at baseline, after 1 and 2 weeks. * *p* < 0.05. One-way ANOVA followed by Tukey’s post-hoc test.

No adverse effects were recorded during the study period. In addition, no side effects have been recorded following the administration of the product Test and Control (Figure 2, Figure 3 and Figure 4). At the end of the treatment cycle, the patients underwent finishing and polishing for the removal of any extrinsic pigmentation on the teeth. No drop-out was recorded in phase V2, V3, and V4, and all patients were evaluated according to protocol.

### 3.2. Study Outcomes

FMPS score recorded at baseline (V2) were 54.20 ± 10.8 in the Group Test and 58.2 ± 6.1 in the Group Control (*p >* 0.05). After 1 week (V3), FMPS was 13.3 ± 5.6 in the Group Test and 18.7 ± 4.3 in the Group Control (*p < 0*.05), while at V4 (2 weeks), FMPS was 14.2 ± 4.1 in the Group Test and 20.3 ± 5.2 in the Group Control (*p <* 0.05) (Figure 2). The Pearson coefficient revealed a FMPS inter-operator r: 0.849 (Figure 2).

FMBS score recorded at baseline (V2) was 46.7 ± 8.7 in the Group Test and 49.2 ± 6.2 in the Group Control (*p >* 0.05) (Figure 3). After 1 week (V3), FMBS was 12.7 ± 4.2 in the Group Test and 18.5 ± 5.9 in the Control Group (*p <* 0.05), while after 2 weeks (V4), it was 13.1 ± 3.2 in the Group Test and 19.8± 4.9 Group Control (*p <* 0.05) (Figure 3). The Pearson coefficient showed a FMBS inter-operator r: 0.958 (Figure 3).

The Gingival Index data measured at different experimental times, at Baseline, 1 week, and 2 weeks, are presented in Figure 4. The gingival index score (GI) recorded at baseline (V2) was 2.75 ± 0.17 in Group Test and 2.71 ± 0.11 in the Group Control (*p >* 0.05). After 1 week (V3), FMPS was 1.14 ± 0.55 in the Group Test and 1.75 ± 0.49 in the Group Control (*p <* 0.05), while at V4 (2 weeks), FMPS was 1.09 ± 0.44 in the Group Test and 1.96 ± 0.39 in the Group Control (*p <* 0.05). The Spearman coefficient reported a GI inter-operator r: 0.874 (Figure 3).

## 4. Discussion

The oral environment is constantly subjected to a continuous oxidative stress and free-radicals exposure determined by the tissue inflammation [25,26]. The mouth chronic diseases are able to produce an unbalanced relationship between these exogenous agents and the physiological mechanisms for damage repair [27,28,29]. Several studies demonstrated that the hydrolyzed DNA is able to absolve a protective agent against the oxidative stress in the oral environment [21,30]. Moreover, this component presents a high versatility and could be combined with anti-microbial agents (chlorhexidine, essential oils) in form of topical gels and mouthwash solution [6,18]. Thellung et al., investigated the effect of polydeoxyribonucleotides on human fibroblast cells cultures [31]. The study findings evidenced a dose-dependent response and a significant increase of the proliferation and activity of the fibroblast cells due to the activation of A2 purinergic receptors [31]. The repair action could be supposed as a synergic activity of this pathway with different growth factors, such as epidermal growth factor (EGF), platelet-derived growth factor (PDGF), and fibroblast growth factor (FGF) [32]. In the present investigation, the baseline values showed no statistically significant differences in FMPS and FMBS in the Test and Placebo study groups, both of which exceeded 20%, which, in the literature, is associated with a high risk of progression of periodontal disease [20,22,33,34]. The rationale of the full mouth plaque and bleeding score is determined by the assessment of the mesial, distal, buccal, and palatal/lingual parts of the teeth by a dichotomous measurement (if present or absent) [35,36,37]. These scores take advantage from a rapid assessment, while their limits are correlated to a lower site-specific information about plaque and bleeding distribution [38,39,40]. On the contrary, the bleeding-on probing (BoP) assessment is more accurate for this purpose as a risk progression indicator, but others critical variables could be introduced by this protocol [41]. In fact, the assessment could be influenced by the using of excessive probing forces producing an increase of the risk of false positivity, especially in subjects with thin gum biotypes [40,42,43]. At this scope, the use of calibrated probes in shape and size could also be necessary, but not exhaustive, to standardize the clinical measurements in order to avoid an overestimation and a strong accuracy bias for research purpose. The active phases of periodontal disease are characterized by inflammation of the gingival tissues accompanied by an involvement of the deep structures with destruction of the alveolar bone component and periodontal defects [44,45]. There is evidence from the literature that the control of local factors, such as bacterial plaque, the maintenance of adequate daily oral hygiene from tartar deposits, acquires a central role in counteracting the proliferation of bacterial biofilms and to maintaining a good state of oral health and of periodontal soft tissues [46,47,48,49,50,51,52,53]_._ Although there is little magnitude of difference between the test and control groups (*p* < 0.05), the clinical significance of the study findings should be considered in relation to the not obvious study design that did not consider a surgical or nor/surgical treatment of the subject affected by chronic periodontitis, that represent the main etiological approach to improve the health status of the gingival soft tissues [54]. On the contrary, the present study design limits are correlated to the absence of a long-term effectiveness of the tested mouthwash solution on a wider sample population, while the present research presented a short-term period and single-center trial. As reported previously, the 0.2% CHX-based mouthwash solutions are generally administered for a maximum treatment duration of two weeks, in order to take advantage from an optimal therapeutic antiseptic property, reducing the eventual adverse effects on hard and mucosal soft tissues. In line with these aspects, the rationale of the present investigation was to evaluate only the early-term effect on the periodontal disease status and plaque control findings with no confounding factors related to mechanical/surgical therapy. Moreover, the tested solution could be compared with a wider range of mouthwash formulation producing a comparative analysis on their early inflammation control in chronic periodontitis. Other limits are correlated to the selectivity of the exclusion criteria according to the smoke and the alcohol consumption, in order to produce a more homogeneous study population. At experimental time V3, there is a statistically significant difference between the test group and the placebo group for the parameter FMPS; evidence that is confirmed at two weeks of treatment, in which the average levels of FMPS remain below 20% in the Test Group. Chlorhexidine (CHX) is now known for its optimal antimicrobial and antiplaque properties and its use is considered the Gold Standard for antibacterial action in the oral hygiene [20,55,56]. Ionescu et al., reported in a previous in vitro study on human oral epithelium that the association of CHX with Sodium DNA induced a significantly increased cells viability if compared to the PBS control group with a lower number of dead cells [21]. In this way, the authors reported that the sodium-DNA suggests a protection capability against cellular degeneration correlated to the oxidative stress and the exposure to chlorhexidine solution [21]. The povidone iodine combined to chlorhexidine has been reported in the literature as a useful tool in many different applications, such as preoperative asepsis of the skin, to prevent intravascular-catheter-related infections and to promote the repair of oral wounds with a topical solution [57,58,59]. Sharma et al., demonstrated in a double-blind study that the CHX and PVP mouthwash by significant decrease in the levels of pro-inflammatory cytokines, IL-2, and IFN-ɣ in subjects affected by chronic gingivitis. The same study underlined that the sustain of the gingivitis is correlated to a Th1 cell-mediated response and overexpression of IL-2 and IFN-ɣ [60]. Conversely, the periodontal diseases are correlated to associated to a Th2 cell-mediated and an overexpression of the IL-4, IL-5, IL-6, and IL-10 mediators [60]. The clinical benefits on periodontal tissues derived from the association of mouthwash with 0.2% chlorhexidine with PVP/VA and Sodium DNA to oral hygiene maintenance maneuvers are also evident in relation to the indices of the FMBS and the Gingival Index which show a variation statistically significant of clinical parameters. The single components effect has been already investigated in literature, but the combination represents the novelty of the present study. Moreover, the introduction of other arms in the study design could be a key point for further investigation.

## 5. Conclusions

A significant reduction of inflammation and bleeding scores was found in periodontal patients treated by a mouthwash containing Chlorhexidine 0.2% with Anti Discoloration System (ADS), PVP-VA, and Sodium DNA compared to those treated with a placebo mouthwash. The study findings reported that the novel formulation of chlorhexidine mouthwash showed its efficacy in bacterial plaque reducing and gingival inflammation.

## Figures and Tables

**Table 1:** Pocket depth assessment of the Test and Control Group at the baseline and after 2 weeks.

Pocket Depth Grade	Test Group- Baseline	Test Group-Post Treatment (2 Weeks)	Control Group- Baseline	Control Group- (2 Weeks)
1–2 mm	29.70%	32.50%	37.30%	37.10%
3–4 mm	44.30%	41.60%	31.90%	32.40%
5–6 mm	21.70%	21.50%	27.40%	27.20%
>6 mm	4.30%	4.40%	3.40%	3.30%

**Table 2:** FMPS, FMBS, and Gingival Index assessment of the Test and Control Group at the baseline, after 1 week, and 2 weeks.

FMPS (Mean, SD)	Test Group	Control Group	*p* Value
Baseline	52.7% ± 9.2% [95%CI: −64.20/169.6]	58.2% ± 6.1% [95%CI: −19.31/135.7]	*p* = 0.517
1 week	13.3% ± 5.6% [95%CI:−57.85/84.45]	18.7% ± 4.3% [95%CI: −35.94/73.34]	*p* = 0.031
2 weeks	14.2% ± 4.1% [95%CI: −37.90/66.30]	20.3% ± 5.2% [95%CI: −45.77/86.37]	*p* = 0.046
**FMBS** **(mean, SD)**	**Test Group**	**Control Group**	***p* value**
Baseline	46.7% ± 8.7% [95%CI: −63.84/157.2]	49.2% ± 6.2% [95%CI: −29.58/128.0]	*p* = 0.731
1 week	12.7% ± 4.2% [95%CI: −40.67/66.07]	18.5% ± 5.9% [95%CI: −56.47/93.47]	*p* = 0.026
2 weeks	13.1% ± 3.2% [95%CI: −27.56/53.66]	19.8% ± 4.9% [95%CI: −42.46/82.06]	*p* = 0.041
**Gingival Index** **(mean, SD)**	**Test Group**	**Control Group**	***p* value**
Baseline	2.75% ± 0.17% [95%CI: −3.122/8.822]	2.71% ± 0.11% [95%CI: −3.770/9.190]	*p* = 0.937
1 week	1.14% ± 0.55% [95%CI: −5.848/8.128]	1.75% ± 0.49% [95%CI: −4.476/7.976]	*p* = 0.046
2 weeks	1.09% ± 0.44% [95%CI: −4.501/6.681]	1.96% ± 0.39% [95%CI: −2.995/6.915]	*p* = 0.038

## Data Availability

All experimental data to support the findings of this study are available contacting the corresponding author upon request.

## Abbreviations

***CHX***	Chlorexidine
***PVP/VA***	Polyvinylpyrrolidone/vinyl acetate (PVP-VA)
***ADS***	Anti-discoloration System
***FMPS***	Full Mouth Plaque Score
***FMBS***	Full Mouth Bleeding Score
***GI***	Gingival Index
***V1***	First Visit
***V2***	Visit at the baseline
***V3***	Visit after 2 weeks
